# Active immunization with norovirus P particle-based amyloid-β chimeric protein vaccine induces high titers of anti-Aβ antibodies in mice

**DOI:** 10.1186/s12865-019-0289-9

**Published:** 2019-02-12

**Authors:** Ping Yang, Yongqing Guo, Yao Sun, Bin Yu, Haihong Zhang, Jiaxin Wu, Xianghui Yu, Hui Wu, Wei Kong

**Affiliations:** 1 National Engineering Laboratory for AIDS Vaccine, School of Life Sciences, Jilin University, Changchun, 130012 China; 2 Key Laboratory for Molecular Enzymology and Engineering, the Ministry of Education, School of Life Sciences, Jilin University, Changchun, 130012 China

**Keywords:** Alzheimer’s disease, Norovirus P particle, Amyloid-β, Protein vaccine, Active immunization

## Abstract

**Background:**

Active immunotherapy targeting amyloid-β (Aβ) is a promising treatment for Alzheimer’s disease (AD). Numerous preclinical studies and clinical trials demonstrated that a safe and effective AD vaccine should induce high titers of anti-Aβ antibodies while avoiding the activation of T cells specific to Aβ.

**Results:**

An untagged Aβ1–6 chimeric protein vaccine against AD based on norovirus (NoV) P particle was expressed in *Escherichia coli* and obtained by sequential chromatography. Analysis of protein characteristics showed that the untagged Aβ1–6 chimeric protein expressed in soluble form exhibited the highest particle homogeneity, with highest purity and minimal host cell protein (HCP) and residual DNA content. Importantly, the untagged Aβ1–6 chimeric soluble protein could induce the strongest Aβ-specific humoral immune responses without activation of harmful Aβ-specific T cells in mice.

**Conclusions:**

The untagged Aβ1–6 chimeric protein vaccine is safe and highly immunogenic. Further research will determine the efficacy in cognitive improvement and disease progression delay.

**Electronic supplementary material:**

The online version of this article (10.1186/s12865-019-0289-9) contains supplementary material, which is available to authorized users.

## Background

Alzheimer’s disease (AD) is the most common cause of dementia, contributing mainly to cognitive impairment and memory loss in elderly population [1]. According to the World Alzheimer Report 2016, an estimated 47 million people suffer from dementia worldwide, and this number is expected to increase to more than 131 million by 2050 [2]. Because of the aging population, the prevalence and incidence of AD in the United States, Australia, Asia, Europe, and the world as a whole are increasing annually, with considerable social and economic burdens [3–9]. In Mainland China, Chan et al. determined that the number of people with AD was 1.93 million in 1990 and 5.69 million in 2010; the incidence of dementia was 9.87 cases per 1000 person-years, while that of AD was 6.25 cases per 1000 person-years, which was significantly higher than that estimated in the World Alzheimer Report 2009 [10, 11]. Currently, only four acetylcholinesterase (AChE) inhibitors (AChEIs) and one N-methyl D-aspartate (NMDA) receptor antagonist—memantine—have been approved for the management of cognitive symptoms of AD [12]. However, these treatments do not prevent the progression of the disease. Therefore, an effective therapy to halt the progression of AD is urgently needed.

According to the amyloid cascade hypothesis, the presence of large numbers of “senile plaques” in the brain resulting from the deposition of β-amyloid (Aβ) peptides plays an important role in AD pathogenesis [13–17]. Both active and passive anti-Aβ immunotherapy for AD have been developed, and data from preclinical studies and clinical trials indicate that high titers of Aβ-specific antibodies may prevent the aggregation of toxic forms of Aβ peptides and can be beneficial to AD patients, suggesting that induction of Aβ clearance may be a promising therapeutic approach to delay the course of this disease [18–25].

Induction of high levels of Aβ-specific antibodies is essential for the clearance of Aβ plaques in the brain; however, as a safe hapten, Aβ1–6 itself is not immunogenic. It must be coupled with an ideal carrier to induce high levels of antibodies. Human norovirus (NoV), also known as Norwalk-like virus, is responsible for most epidemic outbreaks of gastroenteritis. The NoV capsid protein contains two major domains, the shell (S) and protruding (P) domains [26]. Expression of the P domain in vitro spontaneously results in the formation of different P domain complexes: the P dimer, the 12-mer small P particle, and the 24-mer P particle [27–30]. Each P domain has three surface loops that can be used for foreign antigen presentation; therefore, a 24-mer P particle can present 72 copies of antigens, which could efficiently enhance the immunogenicity of the antigens [31–35]. Thus, the P particles may be an excellent platform for vaccine development and antibody production against AD.

We previously demonstrated that a recombinant Aβ1–6 chimeric protein vaccine with His-tag, comprising three copies of Aβ1–6 inserted into the three loops of the NoV P particle, was immunogenic and protective in a mouse model of AD [36, 37]. However, due to some of the disadvantages of tagged protein, it is not suitable for future clinical application. First, the tag may negatively affect the protein conformation, bioactivity and function, and the removal of the tag is a crucial step particularly in cases when the target protein is intended for pharmaceutical or therapeutic applications in addition to crystallization and structural determination. Second, though the affinity tag could be removed by enzyme cleavage, but the recovery rate, the residual enzyme and tag were hard to control and detect during the process. Most importantly, the protein structure and function may change during the process [38–42].

In the present study, the objective was to evaluate the characteristics and immunogenicity of an untagged Aβ1–6 chimeric protein vaccine based on NoV P particle that was more suitable for clinical use. For the first time, we expressed a recombinant Aβ1–6 chimeric protein without affinity tag in *Escherichia coli*, purified it through a series of chromatographic methods, and demonstrated its efficacy in inducing high titers of Aβ-specific antibodies in vivo. In addition, we also investigated the levels of Aβ-specific antibodies generated by Aβ1–6 chimeric soluble protein (SP) and inclusion bodies (IB).

## Results

### Purification of recombinant untagged Aβ1–6 chimeric protein

To increase the expression of untagged Aβ1–6 chimeric protein in the soluble form, several culture conditions were tested. Initially, the induction time, temperature, and addition of IPTG were analyzed (data not shown). The protein was most efficiently produced after 16–18 h of induction with 0.1 mM IPTG at 16 °C (data not shown).

**Fig. 1:**
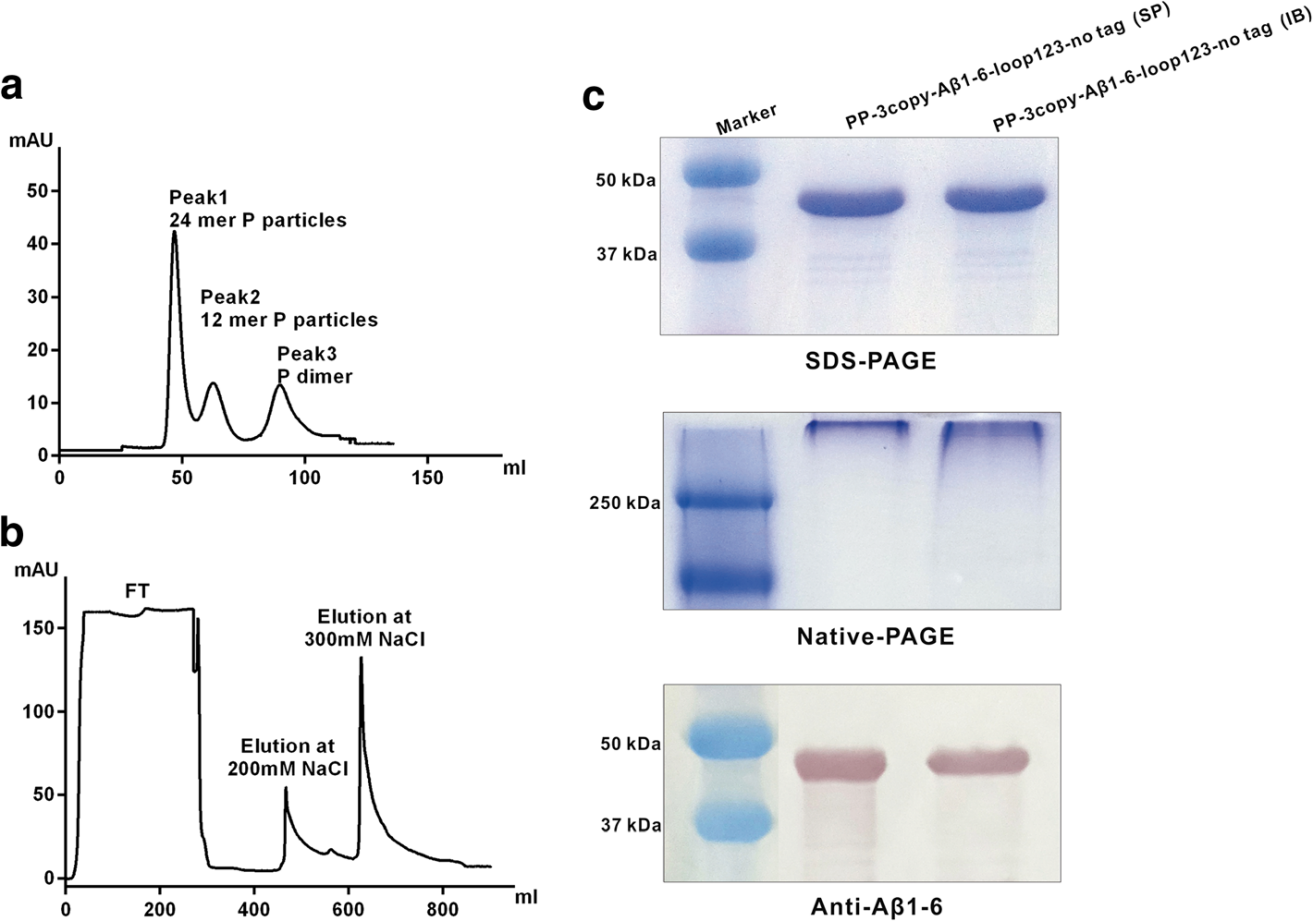
Production and purification of untagged Aβ1–6 chimeric protein (**a**) Size-exclusion chromatography of the untagged Aβ1–6 chimeric SP using a Superdex 200 prep grade column. (**b**) IB purified by DEAE anion-exchange chromatography. (**c**) SDS-PAGE, Native-PAGE, and anti-Aβ1–6 western blot analysis of SP and IB of untagged Aβ1–6 chimeric proteins

To purify the untagged Aβ1–6 chimeric SP, NaCl buffer (20 mM phosphate-buffer, 1 M NaCl, pH 8.0) was added to the clarified supernatant of the *E. coli* cell lysate. As shown in Fig. 1, the molecular weight of the purified untagged Aβ1–6 chimeric protein was about 44 kDa, which was consistent with the theoretical molecular mass of 43.8 kDa. SDS-PAGE showed that the purity was > 90%. The untagged Aβ1–6 chimeric protein was confirmed by western blot analysis using anti-Aβ1–6 rabbit polyclonal antibody. Further analysis using Superdex 200 gel filtration showed that more than one peak appeared after the protein flow through, indicating that the untagged SP contained multiple types of P particle complexes. Importantly, the major peak appeared at the first peak, which was mainly 24-mer P particle.

For the untagged Aβ1–6 chimeric IB, the major peak appeared when eluted with NaCl at a concentration of 200 mM and 300 mM after loading on the DEAE Sepharose column. SDS-PAGE revealed that the purity was > 90%.

### Purification of recombinant His-tagged Aβ1–6 chimeric protein

**Fig. 2:**
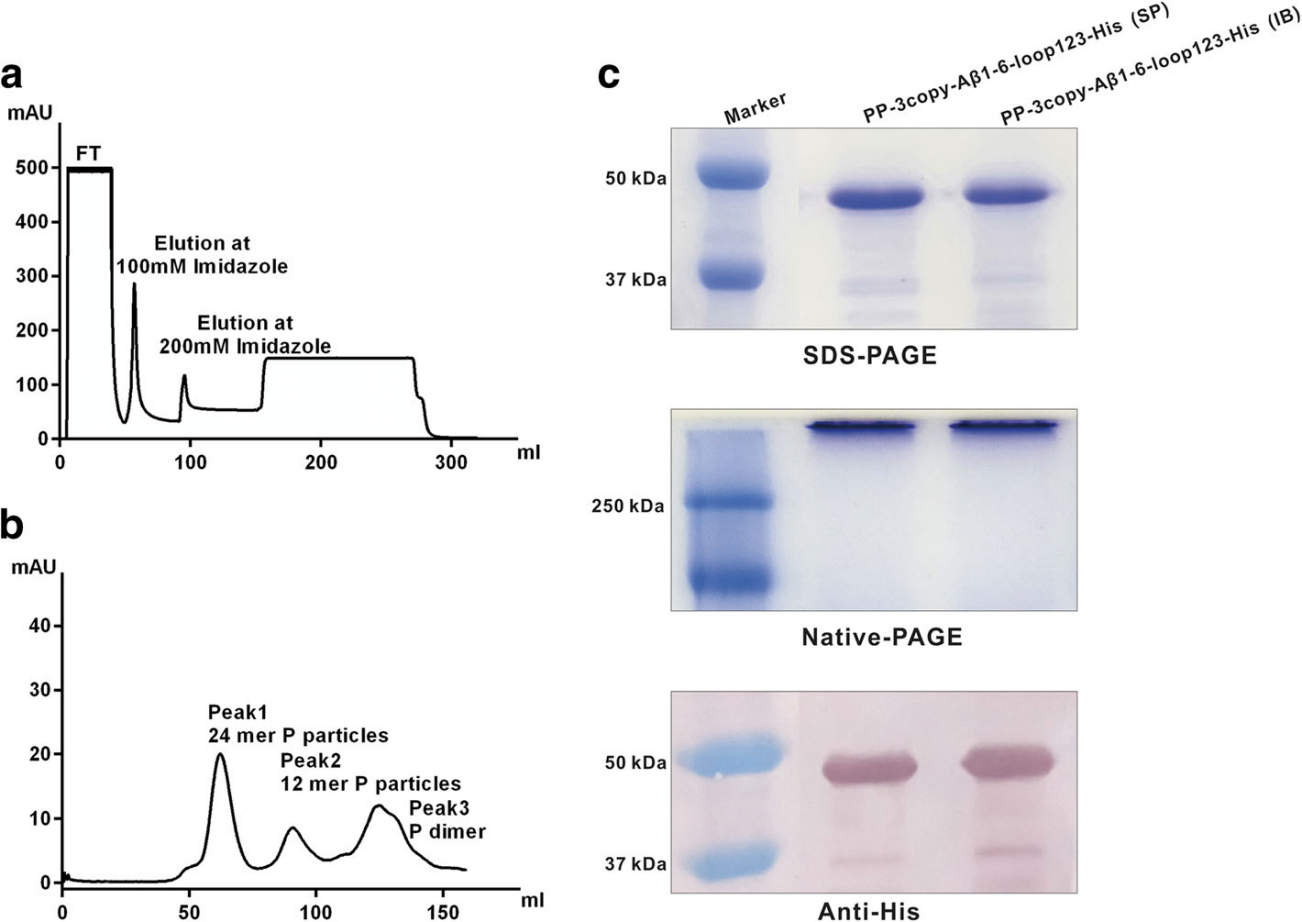
Production and purification of His-tagged Aβ1–6 chimeric protein. (**a**) Ni-NTA affinity purification of the His-tagged Aβ1–6 chimeric SP. Target protein was eluted with 200 mM imidazole. (**b**) Size-exclusion chromatography of the His-tagged Aβ1–6 chimeric SP using a Superdex 200 prep grade column. (**c**) SDS-PAGE, Native-PAGE, and anti-His-tag western blot analysis of SP and IB of His-tagged Aβ1–6 chimeric proteins

As shown in Fig. 2, SDS-PAGE showed the main band was His-tagged Aβ1–6 chimeric protein, and the particle forms were determined by Native-PAGE. Western blot analysis confirmed that the His-tagged protein was recognized and detected by anti-His-tag mouse monoclonal antibody.

### Size analysis of recombinant Aβ1–6 chimeric proteins

**Fig. 3:**
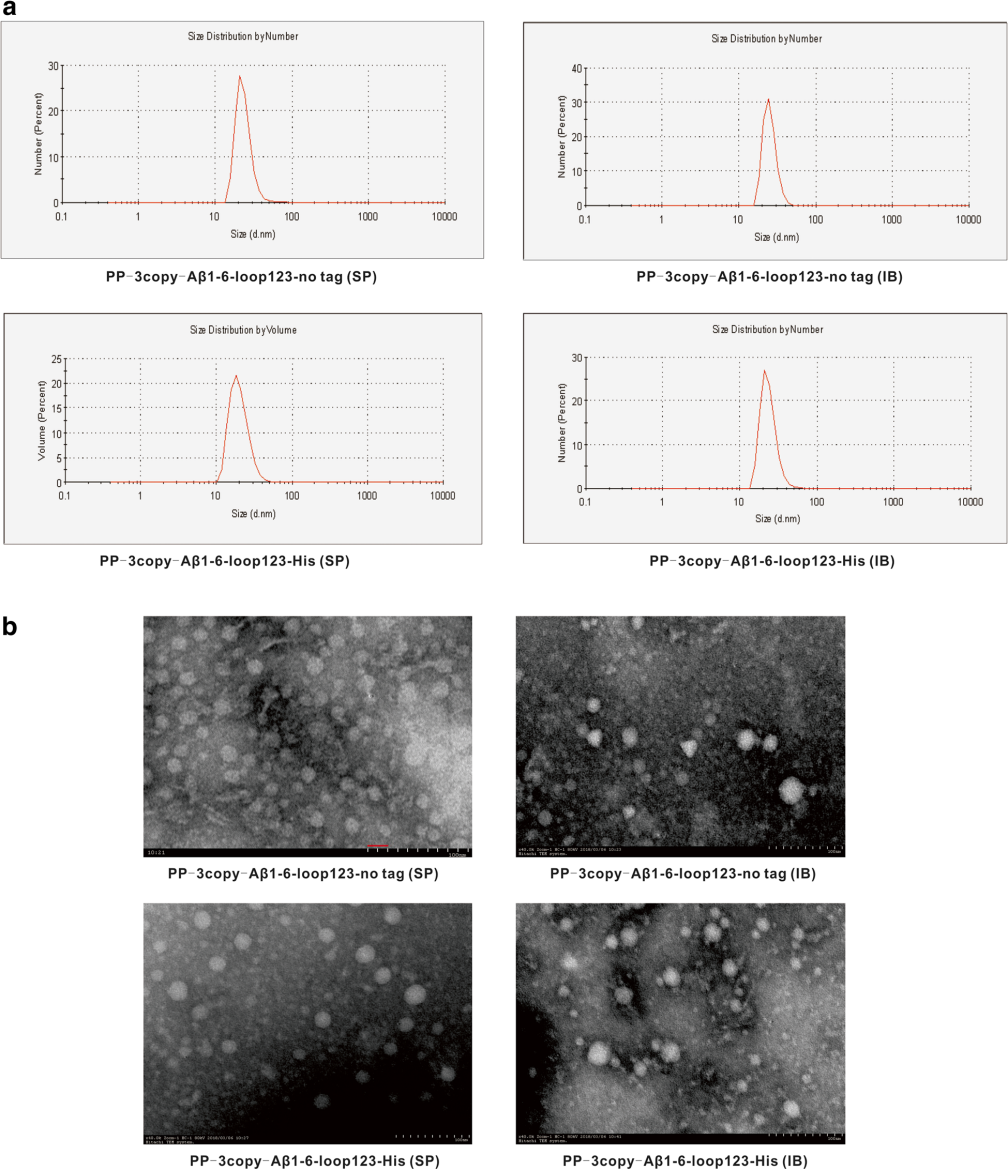
Size analysis of Aβ1–6 chimeric proteins (**a**) DLS detection and (**b**) TEM observation of the SP and IB of the untagged and His-tagged Aβ1–6 chimeric proteins

As shown in Fig. 3, the DLS results demonstrated that the size of untagged Aβ1–6 chimeric SP was 21.78 nm, which was consistent with the particle size measured by TEM, suggesting that the untagged Aβ1–6 chimeric SP mainly forms 24-mer particles. Furthermore, the untagged Aβ1–6 chimeric SP had the highest particle abundance when the same amount of proteins was observed by TEM. Using anion-exchange chromatography, the untagged Aβ1–6 chimeric IB was successfully purified with a particle size of 23.83 nm. The average diameter of purified His-tagged Aβ1–6 chimeric SP and IB was 20.28 nm and 21.22 nm as measured by DLS and TEM.

All P particles appeared to be globular based on TEM observation. Notably, the homogeneity and abundance of the untagged Aβ1–6 chimeric SP was significantly higher than that of other proteins.

### HCP and residual DNA analysis

**Fig. 4:**
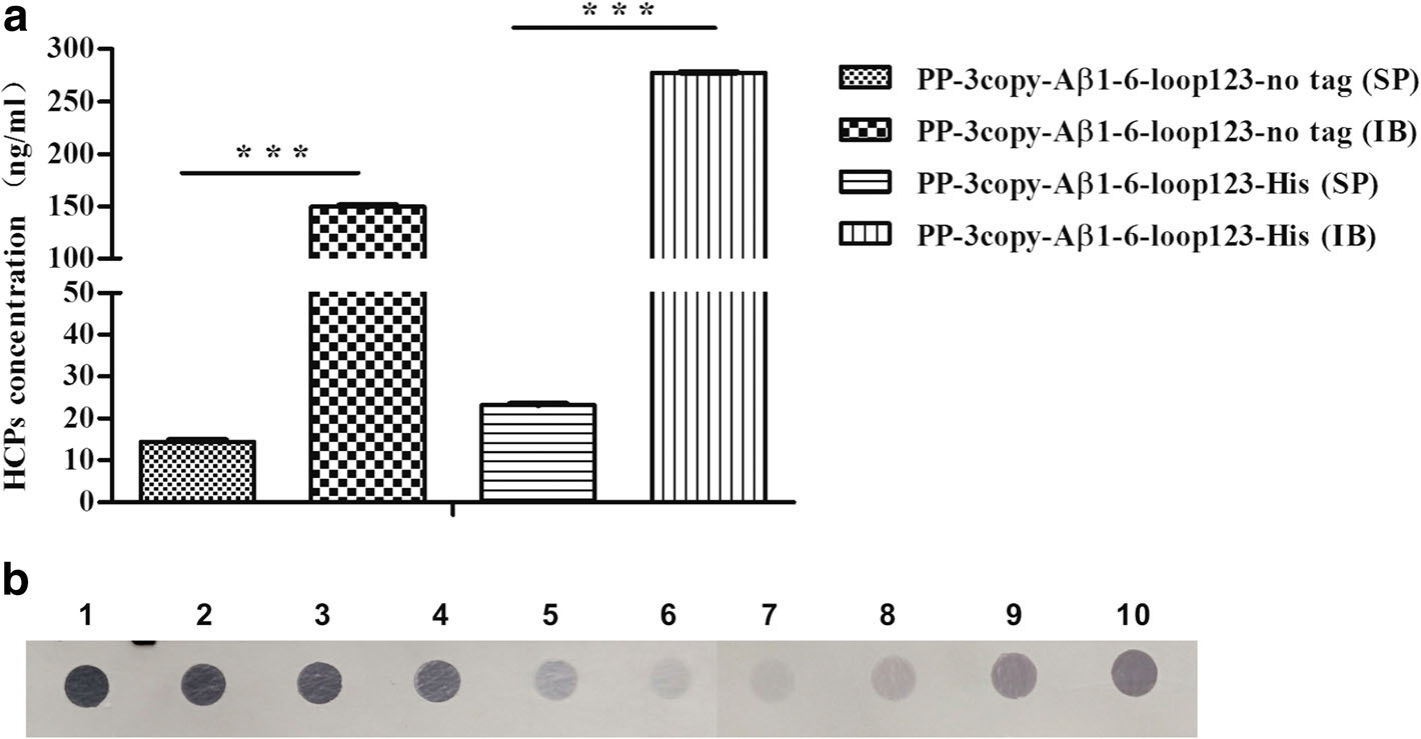
Analysis of HCPs and residual DNA in final products (**a**) HCP detection by ELISA. ***P < 0.001. (**b**) Residual host cell DNA analysis by southern blot. Lanes 1–6: 10 ng, 1 ng, 500 pg, 250 pg, 100 pg, and 20 pg of BL21 (DE3) template DNA. Lanes 7–8: SP of untagged and His-tagged Aβ1–6 chimeric proteins. Lanes 9–10: IB of untagged and His-tagged Aβ1–6 chimeric proteins

To determine if the content of host cell-derived proteins and DNA in the final products conformed to quality requirements, HCPs and residual DNA were detected by ELISA and southern blot, respectively. The results showed that the majority of HCPs were removed by the gel filtration chromatography step. However, during the process of dialysis, it was difficult to remove the HCPs. The concentrations of HCPs in the final samples of IB (untagged and His-tagged) were significantly higher (*P* < 0.001) than in the final samples of SP (Fig. 4a). In addition, the concentrations of residual DNA in the final products of SP (untagged and His-tagged) were < 100 pg/ dose, which was significantly lower than the concentrations of residual DNA in the final products of IB (Fig. 4b).

### Aβ-specific antibody responses

**Fig. 5:**
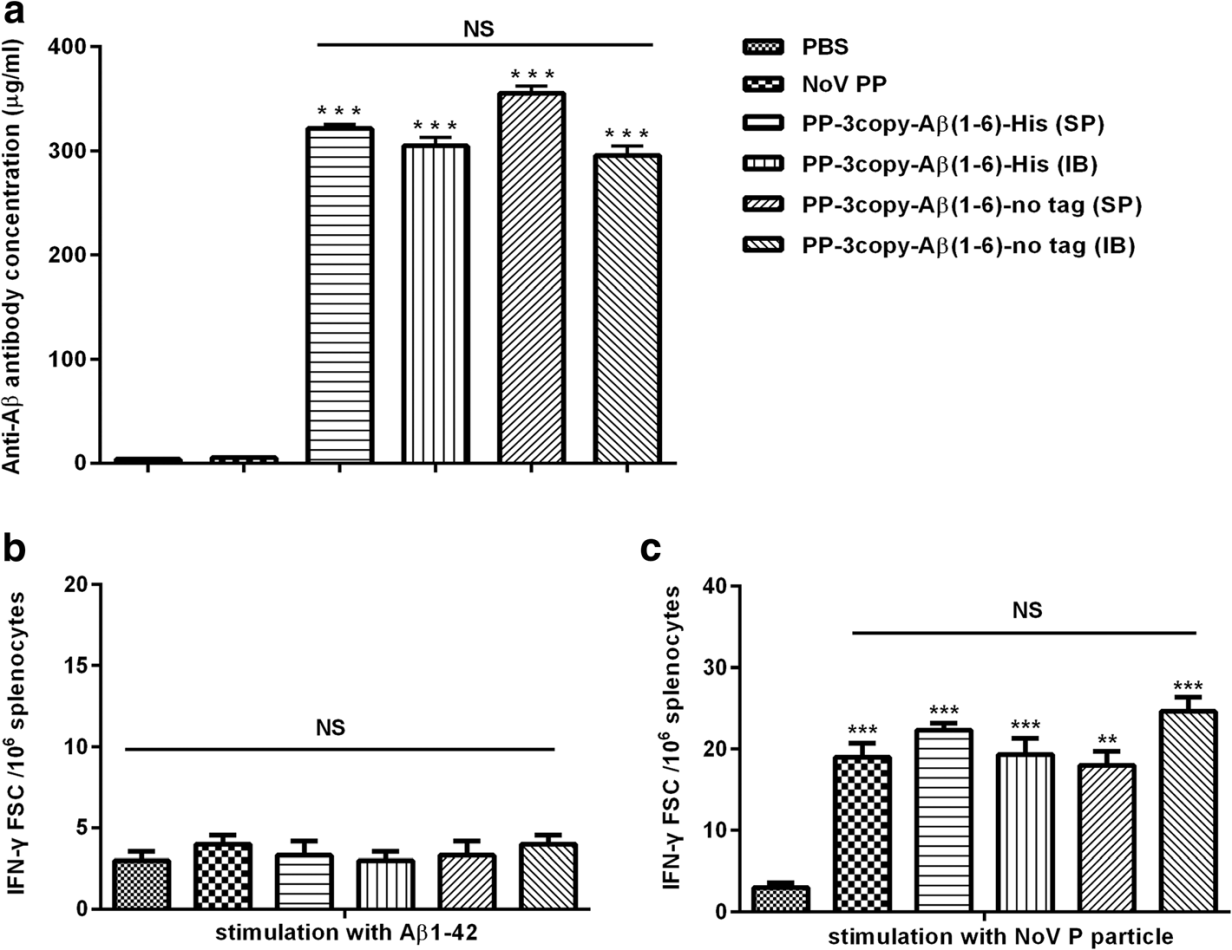
Detection of anti-Aβ antibodies by ELISA and determination of Aβ-specific T cell responses by ELISPOT (**a**) Serum antibody concentrations of mice immunized four times with four types of Aβ1–6 chimeric protein vaccines were detected by ELISA. Serum antibody concentrations were relatively determined according to the standard curve based on 6E10. (**b**) Spleen cells isolated from immunized mice were stimulated with Aβ1–42 peptide. (**c**) Spleen cells were stimulated with wild-type NoV P particles. **P < 0.01, ***P < 0.001, NS = no significant difference

In order to investigate whether the untagged Aβ1–6 chimeric protein vaccine could elicit high titers of Aβ-specific antibodies and determine the ideal expression form of protein vaccine, the immunogenicity of the untagged Aβ1–6 chimeric protein vaccines was evaluated in C57/BL6 mice. As negative control, mice were immunized with PBS or wild-type NoV P particle; as positive control, mice were immunized with His-tagged Aβ1–6 chimeric proteins. Experimental and control animals received four immunizations, and the anti-Aβ antibody responses were analyzed in pooled sera collected from mice after each injection. The titers of anti-Aβ antibody were measured by ELISA. As shown in Fig. 5a, the untagged Aβ1–6 chimeric protein vaccines were effective in inducing strong humoral immune responses. In comparison, the untagged Aβ1–6 chimeric SP induced the highest levels of anti-Aβ antibodies. No anti-Aβ response was detected in the PBS and wild-type NoV P particle control groups.

### Aβ-specific T cell responses

The Aβ-specific T cell responses were analyzed in spleen cells of immunized mice stimulated in vitro with Aβ1–42 peptide. As shown in Fig. 5b, ELISPOT demonstrated that no Aβ-specific T cell response was detected in any experimental mice (*P* > 0.05). Furthermore, the results of immunization with wild-type NoV P particles and recombinant Aβ1–6 chimeric protein vaccines (untagged and His-tagged) showed a strong activation of the P particle-specific T cell response, which was significantly higher (*P* < 0.01) compared with that in the PBS control group (Fig. 5c). We also examined whether recombinant Aβ1–6 chimeric protein vaccines induced the production of Aβ-specific pro-inflammatory cytokines TNF-α and IL-2 in mice. The results showed that splenocytes from Aβ1–6 chimeric protein vaccines immunized mice failed to secrete TNF-α and IL-2 above the detection limit (Additional file 1: Figure S1, *P* > 0.05).

## Discussion

AD is the most common neurodegenerative disorder and is pathologically characterized by Aβ plaques and neurofibrillary tangles (NFTs) [44]. To date, there is no effective treatment to alter the disease course of AD; however, immunotherapy has recently been considered a potential treatment for AD. Many studies have shown that reducing Aβ production and aggregation or enhancing its removal is a rational approach to treating AD [19, 45–49]. Owing to the fewer doses required, longer-lasting effects, and possibly fewer side effects, Aβ-targeted active immunotherapy remains a promising candidate treatment. Though the results of animal experiments and clinical trials have shown the beneficial effect of active anti-Aβ immunization approaches [50, 51], most clinical trials of immunization against Aβ were halted because no slowing effect on cognitive decline was observed; reasons for failure of anti-Aβ immunotherapy include selection of inappropriate Aβ-specific epitopes, inadequate immunopotentiation of adjuvants or carriers, and inappropriate dose and treatment time [23, 52, 53]. Thus, the goal of this study was to develop a safe, and effective active candidate vaccine against AD for future clinical use.

Previously, the results of treatment with the NoV P particle-based chimeric protein vaccine (containing His-tag) bearing Aβ1–6 fragment suggested that this active immunotherapeutic strategy is effective and safe in wild-type and AD model mice [36, 37]. However, the protein fusion tag was not suitable in the clinical trial; the tag should be cleaved from the final products or removed from the constructs in the case of clinical application. Therefore, in the present study, a NoV P particle-based chimeric protein bearing three copies of Aβ1–6 and lacking a protein fusion tag was generated and expressed in *E. coli*. In order to determine the optimal expression form of recombinant Aβ1–6 chimeric protein, the untagged Aβ1–6 chimeric SP and IB expressed in *E. coli* were prepared by different purification processes. The results indicated that the untagged Aβ1–6 chimeric SP, mainly forming 24-mer P particles, was successfully obtained with a purity > 90%. Most importantly, the untagged Aβ1–6 chimeric protein expressed in soluble form exhibited the optimal particle form and the highest 24-mer abundance. Since the IB contained relatively pure and intact recombinant proteins, the untagged Aβ1–6 chimeric IB was attempted to refold in this study, which was successfully assembled into P particles. However, limited amounts of active protein (particle form) were obtained in the refolding process, and consequently the recovery yields were very low (data not shown). Thus, the untagged Aβ1–6 chimeric protein expressed in soluble form was the optimal candidate vaccine against AD for human use.

In *E. coli* protein expression systems, HCPs accompanied with recombinant proteins can significantly affect vaccine efficacy and immunogenicity [54], and residual host cell DNA may be tumorigenic or infectious in the recipient [55]. Therefore, detection and quantification of HCPs and residual DNA are critical for biopharmaceutical products in accordance with regulatory agency guidelines. As determined by ELISA and southern blot, the amount of HCPs and residual DNA varied in the final products obtained by different purification methods; the majority of HCPs and residual DNA were removed by gel filtration chromatography. These results suggested that the removal of host cell impurities should be monitored in the process development for recombinant protein purification. Among the four protein vaccines, the HCPs and DNA content were minimal in the untagged Aβ1–6 chimeric SP and in line with the requirements of the Chinese Pharmacopoeia.

According to the results of preclinical and clinical trials, a qualified anti-Aβ active immune vaccine should induce sufficient humoral immune response to provide therapeutically effective anti-Aβ antibodies but avoid the undesirable Aβ-specific T cell immune response [25, 56–58]. This can be achieved by the use of antigen that only contains the B cell epitope of Aβ and coupling with an ideal vaccine platform for stimulating Aβ antibody production. Thus, in the current study, we selected Aβ1–6 as the antigen and presented it by NoV P particle to generate effective humoral immune responses. NoV P particle predominantly induced the Th2 immune response, which is considered safe and necessary for anti-Aβ immunotherapy [59]. C57/BL6 mice were immunized with untagged Aβ1–6 chimeric protein vaccines by the intramuscular route, and the Aβ-specific antibody and T cell immune responses were compared. We also compared the immune effects of untagged Aβ1–6 chimeric SP and IB in order to determine the optimal expression form of the recombinant protein. As expected, the untagged Aβ1–6 chimeric SP was the most effective in inducing Aβ-specific antibody. Moreover, no Aβ-specific T cell immune response was observed in vaccine groups. The above results demonstrated that the untagged Aβ1–6 chimeric protein vaccine may serve as an effective and safe form of active immunotherapy for AD and thus merits further and more detailed study.

## Conclusion

We successfully developed an untagged NoV P particle-based Aβ1–6 chimeric protein vaccine. This vaccine elicited therapeutically high titers of anti-Aβ antibody without activation of Aβ-specific T cell response. Therefore, based on the data presented here in addition to the considerable potential for cognitive capacity improvement and disease progression delay in APP/PS1 transgenic mice demonstrated in previous studies, the untagged Aβ1–6 chimeric protein vaccine may be a promising candidate vaccine for AD. In the next study, we will confirm its high immunogenicity in the AD model mice and subsequently initiate preclinical testing in non-human primates.

## Methods

### Strains and plasmids

Chemically competent *Escherichia coli* strain DH10b (ThBio, China) was used for plasmid amplification. Competent *E. coli* BL21 (DE3) (TransGen Biotech, China) was used for recombinant protein expression. The pET-26b plasmid vector (Invitrogen, USA) was used to clone the NoV P particle and Aβ1–6 epitope gene fragments.

### Construction of expression vector

A novel NoV P particle-based Aβ1–6 chimeric protein without protein fusion tag was named PP-3copy-Aβ1–6-loop123-no tag and constructed as follows: A synthesized cDNA fragment encoding the NoV P domain (Hu/GII.4; GenBank: DQ078814.2) was cloned into the bacterial expression vector pET-26b (Invitrogen). Next, three copies of Aβ1–6 (DAEFRH) were inserted into loop 1, loop 2, and loop 3 of the P domain using a GGG linker. In order to improve protein expression, the NoV P domain and Aβ1–6 gene were codon-optimized. The PP-3copy-Aβ1–6-loop123 with His-tag was used as a control and was described in our previous study [36, 37].

### Large-scale protein expression

All protein expression was performed in *E. coli* BL21 (DE3) cells using isopropyl β-D-1-thiogalactopyranoside (IPTG; Invitrogen) as an inducer. The cells were cultured with LB medium supplemented with 50 μg/ml kanamycin (Sigma-Aldrich, USA) at 37 °C. Induction was performed by addition of IPTG to a final concentration of 0.1 mM when the OD_600_ was 0.6–0.8. After culturing for another 16–18 h at 16 °C, cells were harvested by centrifugation at 6000×*g* for 30 min and washed with phosphate-buffered saline (PBS) by centrifugation at 8000×*g* for 15 min. The cell pellet was suspended in lysis buffer (20 mM phosphate-buffer or 50 mM Tris-HCl, pH 8.0) in a beaker maintained on ice, then disrupted with a sonicator programmed for 30 min of 5 s on and 5 s off. Following cell lysis, the homogenates were clarified by centrifugation at 30,000×*g* for 30 min. The soluble protein (SP) in supernatants and the inclusion body protein (IB) in precipitates were further treated by different methods.

### Purification of recombinant Aβ1–6 chimeric SP

The untagged Aβ1–6 chimeric SP was precipitated by adding salt solution (20 mM phosphate-buffer, 1 M NaCl, pH 8.0) and mildly agitating at 4 °C overnight. Centrifugation was performed at 30,000×*g* for 30 min, and then the pellets were resuspended with 20 mM phosphate-buffer (pH 8.0) and mildly agitated at 4 °C for 6–8 h. Next, the supernatants were harvested by centrifugation at 25,000×*g* for 30 min and were concentrated by ultracentrifugation through a 10% (wt/vol) sucrose cushion at 100,000×*g* for 4 h at 4 °C; the pellet was resuspended in a minimum volume (1–2 ml) of PBS buffer. Finally, the concentrates were further isolated by a HiLoad 16/60 Superdex 200 prep grade column (GE Healthcare, USA). Samples were detected by sodium dodecyl sulfate-polyacrylamide gel electrophoresis (SDS-PAGE), Native-PAGE, and western blot assays (primary antibody: anti-Aβ1–6 rabbit polyclonal antibody, AnaSpec, CA).

The His-tagged Aβ1–6 chimeric SP was prepared according to our previous study [43] and used as a positive control. Samples were detected by SDS-PAGE, Native-PAGE, and western blot assays (primary antibody: anti-His-tag mouse monoclonal antibody, Thermo Fisher Scientific, USA).

### Refolding of recombinant Aβ1–6 chimeric IB

The untagged Aβ1–6 chimeric IB was dissolved in 8 M urea buffer (50 mM Tris-HCl, 0.1 M NaCl, pH 8.0) and refolded in refolding buffer (50 mM Tris-HCl, 0.1 M NaCl, 1 mM EDTA,1% Glycine, 5% Glycerol, pH 8.0,) through a typical dialysis procedure, then applied to anion-exchange chromatography column (DEAE Sepharose; GE Healthcare) pre-equilibrated with buffer A (50 mM Tris-HCl, 0.1 M NaCl, pH 8.0) and eluted with a gradient of NaCl from 100 to 500 mM in buffer B (50 mM Tris-HCl, 1 M NaCl, pH 8.0).

The refolding of His-tagged Aβ1–6 chimeric IB has been described previously [43]. Final samples were detected by SDS-PAGE, Native-PAGE, and western blot assays.

### Analysis of particle size

According to our previous study [43], the size of Aβ1–6 chimeric proteins was analyzed by dynamic light scattering (DLS) and transmission electron microscopy (TEM).

### Analysis of contaminant host cell-derived proteins and DNA

During expression of a recombinant protein, the host cell system can express many endogenous proteins, and residual DNA is inevitably present in the final products. An *E. coli* host cell protein (HCP) enzyme-linked immunosorbent assay (ELISA) kit (#F410, Cygnus Technologies, USA) was used to determine the presence of HCP contamination in the final products, and residual host cell DNA was detected by southern blot assay using a DIG-High Prime DNA Labeling and Detection Starter Kit I (Roche, Switzerland) according to the manual.

### Mouse immunization

Protein concentrations were determined with the Pierce™ BCA protein assay kit (Thermo Scientific, USA) with bovine serum albumin (BSA) as a calibration standard. The SP and IB of NoV P particle-based Aβ1–6 chimeric proteins (untagged and and His-tagged) were used to evaluate the immunogenicity. Adult female C57BL/6 mice (8–9 weeks old) were randomly divided into six groups (*n* = 6 per group) and immunized intramuscularly with 25 μg of Aβ1–6 chimeric protein mixed with 10 μg of CpG oligodeoxynucleotides. In addition, one group of mice received 25 μg of wild-type NoV P particle mixed with 10 μg of CpG oligodeoxynucleotides (ODN). Control mice were inoculated with PBS in the presence of CpG adjuvant. Mice in each group were immunized four times at 2-week intervals. Blood samples were collected from the periocular vein after each vaccination to monitor the immune response. Aβ-specific antibody levels were detected by ELISA. At two weeks after the fourth immunization, mice were euthanized by CO2 inhalation, the spleens were aseptically removed, and single-cell suspensions were prepared for cytokine detection by an enzyme-linked immunospot assay (ELISPOT).

The C57BL/6 mice were obtained from the Changchun Institute of Biological Products Co., Ltd. (Changchun, China). Mice had been housed in clean-grade environment, fed with standard diet, had been allowed ad libitum access to food and water and taken care of on a 12-h light-dark cycle. All animal experiments were approved by the Ethical Committee of Care and Use of Laboratory Animals at Jilin University and performed in compliance with legal and institutional guidelines.

### Antibody ELISA

Aβ-specific antibody responses were assessed by ELISA. Briefly, 96-well microplates were coated with 100 ng Aβ1–42 peptides (GL Biochem, China) per well, incubated overnight at 4 °C, and then blocked for 1 h with PBS containing 5% BSA (Bovogen, Australia). Plates were washed three times and incubated for 2 h at 37 °C with pooled mouse sera. After three washes, plates were incubated with 1:5000-diluted secondary anti-mouse IgG (horseradish peroxidase-conjugated, Jackson Laboratories, UK) for 1 h at 37 °C. Plates were then washed, developed with 3,3′,5,5′-tetramethylbenzidine (TMB) substrate, and examined at 450 nm. For determination of serum antibody concentrations, the mouse Aβ1–16-specific monoclonal antibody 6E10 (1 mg/ml; BioLegend, USA) was used for the calibration curve. Briefly, serum samples and antibody 6E10 were tested in duplicate using fourfold serial dilutions starting at 1:800, and the serum antibody concentrations was relatively calculated according to the standard curve based on 6E10.

### Cytokines assays

Spleens were aseptically removed from euthanized mice, and a cell suspension was obtained by grinding the specimens in ACK lysis buffer (Beyotime, China). Leukocytes (pooled in each group) were resuspended at 1 × 10^7^ cells/ml in Roswell Park Memorial Institute (RPMI) 1640 medium (Gibco, USA) supplemented with 10% fetal bovine serum (FBS) and 100 U of penicillin-streptomycin (Invitrogen). Aliquots (100 μl) of the cell suspension were added to wells of a flat-bottom 96-well plate. Cells were grown in the presence of Aβ1–42 peptides and wild-type NoV P particles. Positive control cultures were stimulated with 2 μg/ml of concanavalin A (Con A). Negative control cultures were grown in RPMI 1640 (10% FBS) medium. Cultures were grown for 24 h at 37 °C under a humidified atmosphere with 5% CO_2_. Cytokine release from stimulated cells was measured using a mouse IFN-γ ELISPOT kit (BD Biosciences, USA) according to the manufacturer’s recommendations. In addition, splenocytes (5 × 10^6^ cells) from immunized C57BL/6 mice were cultured in the presence of Aβ1–42 peptides, positive and negative controls respectively, culture supernatants were harvested after 48 h incubation, and cytokines TNF-α and IL-2 were measured by using mouse TNF-α and IL-2 ELISA kits (BioLegend, USA) according to the assay protocols.

### Statistical analysis

Antibody and T cell immune responses were compared between the different groups with Prism 5 software for Windows (GraphPad Software, USA). The results were expressed as mean values ± standard error of the mean (SEM). Comparisons between groups were analyzed using standard two-tailed unpaired t-tests, and data with a *P*-value < 0.05 were considered statistically significant.

## Additional file

Additional file 1: **Figure S1**. Determination of Aβ-specific cytokines TNF-α and IL-2 by ELISA. (A) Splenocytes isolated from immunized mice were cultured with Aβ1–42 peptide for 48 h, culture supernatants were measured using mouse TNF-α detection kit. (B) Splenocytes were cultured with Aβ1–42 peptide for 48 h, levels of IL-2 in culture supernatants were determined by mouse IL-2 detection kit. NS = no significant difference. (TIF 20623 kb)

## Abbreviations

AD: Alzheimer’s disease
Aβ: β-amyloid
BSA: Bovine serum albumin
DLS: Dynamic light scattering
ELISA: Enzyme-linked immunosorbent assay
ELISPOT: Enzyme-linked immunospot assay
FBS: Fetal bovine serum
HCP: Host cell protein
IB: Inclusion body protein
IPTG: Isopropyl β-D-1-thiogalactopyranoside
NoV: Norovirus
P: Protruding
PBS: Phosphate-buffered saline
RPMI: Roswell Park Memorial Institute
SDS-PAGE: Sodium dodecyl sulfate-polyacrylamide gel electrophoresis
SP: Soluble protein
TEM: Transmission electron microscopy
